# mTOR Signaling: Roles in Hepatitis B Virus Infection and Hepatocellular Carcinoma

**DOI:** 10.7150/ijbs.95894

**Published:** 2024-08-01

**Authors:** Ling Mei, Huizhen Sun, Ying Yan, Huimin Ji, Qian Su, Le Chang, Lunan Wang

**Affiliations:** 1 National Center for Clinical Laboratories, Institute of Geriatric Medicine, Chinese Academy of Medical Sciences, Beijing Hospital/ National Center of Gerontology, Beijing, 100730, P.R. China.; 2 Beijing Engineering Research Center of Laboratory Medicine, Beijing Hospital, Beijing, 100730, P.R. China.; 3 National Center for Clinical Laboratories, Peking Union Medical College, Chinese Academy of Medical Sciences, Beijing, 100730, P.R. China.

**Keywords:** mammalian target of rapamycin, hepatitis B virus, hepatocellular carcinoma, mTOR inhibitors

## Abstract

Currently, chronic hepatitis B virus infection is still one of the most serious public health problems in the world. Though current strategies are effective in controlling infection and slowing down the disease process, it remains a big challenge to achieve a functional cure for chronic hepatitis B in a majority of patients due to the inability to clear the cccDNA pool. The mammalian target of rapamycin (mTOR) integrates nutrition, energy, growth factors, and other extracellular signals, participating in gene transcription, protein translation, ribosome synthesis, and other biological processes. Additionally, mTOR plays an extremely important role in cell growth, apoptosis, autophagy, and metabolism. More and more evidence show that HBV infection can activate the mTOR pathway, suggesting that HBV uses or hijacks the mTOR pathway to facilitate its own replication. Therefore, mTOR signaling pathway may be a key target for controlling HBV infection. However, the role of the central cytokine mTOR in the pathogenesis of HBV infection has not yet been systematically addressed. Notably, mTOR is commonly activated in hepatocellular carcinoma, which can progress from chronic hepatitis B. This review systematically summarizes the role of mTOR in the life cycle of HBV and its impact on the clinical progression of HBV infection.

## Introduction

Hepatitis B virus (HBV) infection remains a major global public health concern, with an estimated 257 million individuals worldwide suffering from chronic HBV infection^1^. Chronic HBV infection can lead to serious progressive liver diseases, such as cirrhosis, liver failure, and hepatocellular carcinoma (HCC)^2^,^3^, which is the third leading cause of cancer-related deaths globally^4^. While effective vaccines have been instrumental in reducing HBV infection rates, particularly in infants, the efficacy of existing medications, including alpha interferon and nucleoside analogues that block viral DNA polymerase is hindered by low rates of sustained response, adverse effects, and the emergence of drug resistance^1^,^5^-^7^. The pathogenesis of HBV infection emphasizes that the specific immune response is not only responsible for viral clearance but also results in hepatocyte inflammatory and regenerative responses. It triggers mitogenic stimuli and mutagenic factors for the formation of DNA damage that can lead to the development of HCC^8^-^12^.

It is worth noting that there is a contradiction between the theory of the pathogenesis of HBV infection and the clinical practice of medication. Currently, the pathogenesis emphasizes that the damage caused by HBV infection is mediated by immunity, while the clinical treatment focuses on inhibiting HBV replication to alleviate liver disease. This incongruity raises questions about the need for additional supplements to the pathogenesis of HBV infection, which could enhance our understanding of the disease and optimize the treatment plans. The mammalian target of rapamycin (mTOR) signaling pathway has been found to regulate the life cycle of many viruses^13^,^14^, and understanding the role of mTOR in the life cycle of HBV is not only significant for clarifying the pathogenesis of HBV, but also for developing effective treatment strategies^15^.

HCC is one of the most common tumors worldwide, and its treatment methods include surgical resection, percutaneous ethanol injection (PEI), radiofrequency ablation (RFA), transcatheter arterial chemoembolization (TACE), and liver transplantation. However, these methods are only suitable for a small number of patients and often lead to postoperative complications^16^. Fewer than 10% of HCC patients are cured, and most eventually progress to advanced HCC. At present, only systemic treatment can effectively delay the natural course of the disease^17^. The current frontline treatment for systemic therapy in clinical practice is the tyrosine kinase inhibitor (TKI) Sorafenib, both the RAF/MEK/ERK pathway and receptor tyrosine kinases to inhibit tumor growth and angiogenesis^18^. However, its median survival is only 10.7 months^19^. Given that mTOR is activated in 40% -50% HCC cases^20^-^23^, it is essential to elaborate on the role of mTOR in the progression of HCC. This review aims to summarize the latest developments in the interaction between HBV and mTOR, as well as the impact of mTOR on HCC progression.

## The structure and function of mTOR

**Figure 1:**
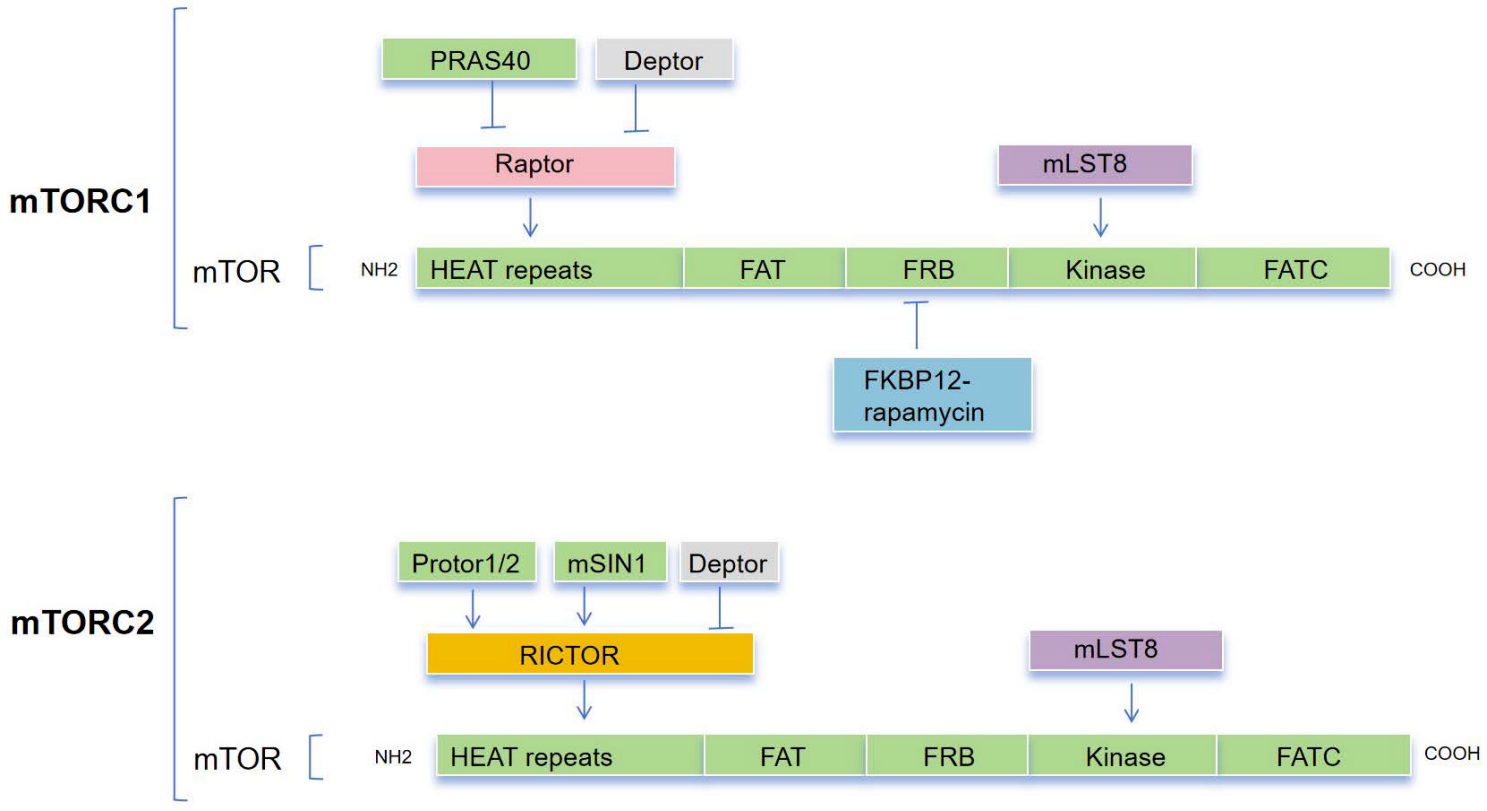
The composition of both mTORC1 and mTORC1. mTORC1 comprises a core of mTOR, mLST8, and Raptor that is suppressed by PRAS40 and DEPTOR. mTORC2 is composed of a basic complex of mTOR, mLST8, and Rictor, which is inhibited by DEPTOR and regulated by mSin1 and Protor1/2. A combination consisting of rapamycin and the cytoplasmic receptor FKBP12 binds to the FRB domain, allosterically inhibiting mTOR action.

The mTOR protein is a member of the phosphoinositide 3-kinase (PI3K)-related kinase (PIKK) family and is an evolutionarily conserved Ser/Thr kinase. it was discovered that mTOR and yeast TOR/DRR proteins, previously identified as rapamycin targets in genetic tests for rapamycin resistance, shared a similarity^24^-^26^. The mTOR protein consists of about 2,500 amino acids and contains functional domains including HEAT repeats, FAT, FRB, Kinase, and FATC (Figure 1). The FAT and FATC domains participate in the interaction between mTOR and its ligands, while the FRB (FKBP12-Rapamycin Binding) domain is involved in the binding of Rapamycin and FKBP12 to inhibit mTOR activity^27^,^28^, and the Kinase domain is responsible for the phosphorylation of downstream targets of mTOR. Overall, the structure of mTOR is intricate, with different domains participating in different functions in signal transduction, protein synthesis, and cellular metabolism^29^.

mTOR is the catalytic subunit of two complexes, mTORC1 and mTORC2^30^ (Figure 1), and their activation and function depend on their subcellular localization 31. These complexes can be distinguished by their specific substrates and activities, auxiliary proteins, and varying sensitivity to rapamycin. Three primary constituents comprise mTORC1: the catalytic subunit mTOR, the regulatory subunit Raptor, and mLST8^32^,^33^. Raptor guarantees appropriate subcellular localization and aids in the recruitment of substrates into the complex. By attaching itself to the catalytic domain, mLST8 keeps the kinase activation loop stable. In addition to these elements, mTORC1 contains two inhibitory subunits: DEPTOR and PRAS40 (Akt substrate). mTORC2 consists of mLST8, mTOR subunit and Rictor, which is insensitive to rapamycin and comparable with Raptor. Together with the other regulatory subunits, mSin1 and Protor1/2, mTORC2 also contains the inhibitory Deptor subunit^34^-^37^.

mTORC1 plays a crucial role in regulating numerous essential cellular processes, including glucose homeostasis, lipid synthesis, and autophagy, by functioning as a sensor for growth factors, pressure, energy status, oxygen, and amino acids^38^,^39^. On the other hand, the mTORC2 complex regulates cell survival and cytoskeletal structure^34^,^35^. It also participates in the transcription factor forkhead box protein O3 (FOXO3) pathway, which controls autophagy^40^. Through a negative feedback loop between the mTORC1 and PI3K-AKT pathways, mTORC1 also controls mTORC2 signaling^41^. Tuberous sclerosis 1 (TSC1) and 2 (TSC2) are key upstream regulators of mTORC1. The GTP-bound form of Ras homolog enriched in the brain (Rheb) directly interacts with mTORC1 and strongly stimulates its kinase activity^42^-^44^. However, TSC1/2 negatively regulates mTORC1 activity by reversing Rheb into its inactive GDP-bound state 45,46. Conversely, mTORC2 is a rapamycin-insensitive companion of mTOR^47^ and can be directly activated by PI3K^48^,^49^. Compared with the mTORC1 pathway, the mTORC2 pathway is much less understood. Its downstream targets include several members of the AGC kinase subfamily, such as Akt, serum and glucocorticoid-induced protein kinase 1 (SGK1) and protein kinase C-a (PKC-a). mTORC2 directly activates Akt by phosphorylating the hydrophobic motif (Ser473) of Akt, which is required for its maximum activation^50^. Akt then regulates cellular metabolism, survival, apoptosis, growth and proliferation through the phosphorylation of several effectors. This involves the classic PI3K-AKT-mTOR pathway.

**Figure 2:**
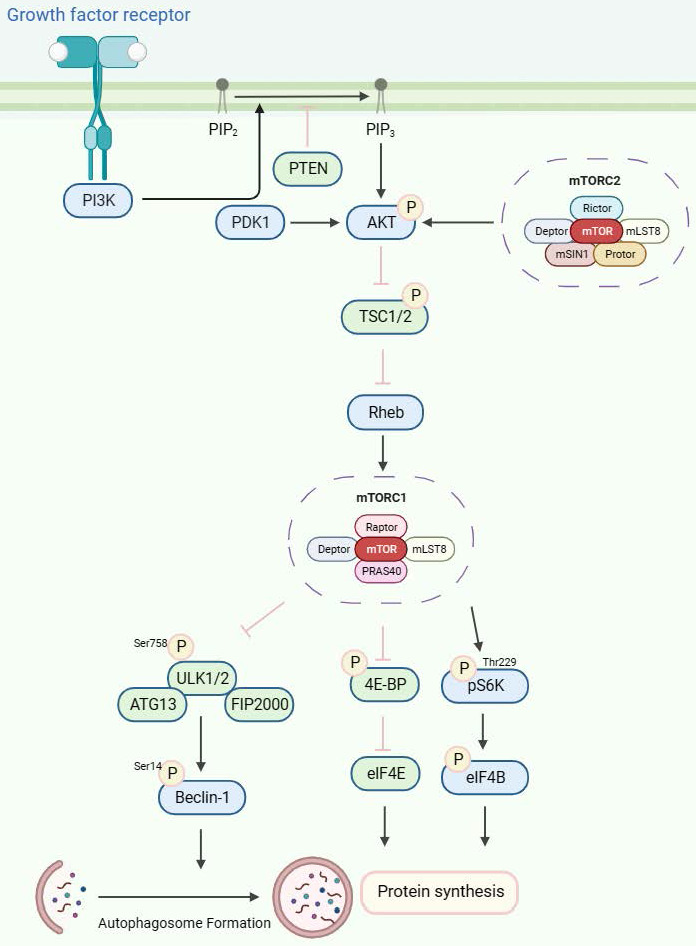
The mTORC1/2 signaling pathways. The receptor tyrosine kinases (RTKs)/PI3K/Akt signaling pathway, stimulated by growth factors, is pivotal to mTOR protein regulation. PI3K, typically maintained at a basal level, is activated to synthesize phosphatidylinositol 3,4,5-triphosphate (PIP3) from phosphatidylinositol 4,5-bisphosphate (PIP2). This process is counteracted by the tumor suppressor PTEN. PIP2 and PIP3 trigger AKT phosphorylation, leading to TSC2 inactivation and Rheb-GTP generation. Upon phosphorylation by mTORC1, ULK1/2 induces autophagy by phosphorylating Beclin-1.

The growth factor-mediated receptor tyrosine kinases (RTKs)/PI3K/Akt signaling pathway is an important upstream signaling pathway for the mTOR protein molecule^51^(Figure 2). Upon stimulation by growth factors, the RTKs initiate signaling cascades that activate PI3K. Generally, PI3K activity is tightly controlled to a basal level under normal conditions. Subsequently, PI3K catalyzes the synthesis of phosphatidylinositol 3,4,5-triphosphate (PIP3) by phosphorylating phosphatidylinositol 4,5-bisphosphate (PIP2). This process is antagonized by phosphatase and tensin homolog deleted on chromosome 10 (PTEN), a tumor suppressor that converts PIP3 to PIP2^52^. PIP2 and PIP3 directly interact with the pleckstrin homology (pH) domain of AKT^53^-^55^, resulting in its phosphorylation by PDK1 at Thr308^56^-^58^. Additionally, the phosphorylation of AKT on Ser473 by mTORC2 is also necessary for its activity^50^,^59^. TSC2 undergoes inactivation through Akt-dependent phosphorylation, which destabilizes TSC2 and disrupts its interaction with TSC1^60^, and acts as a GTPase-activating protein (GAP) complex toward the GTPase RAS homolog enriched in the brain (Rheb)^61^. The mTORC1, a direct target of Rheb-GTP, activates the TOR kinase^62^. The mobilized mTORC1 then relays signaling by phosphorylating two key substrate protein molecules, p70S6K, and 4E-binding protein1 (4EBP1), resulting in activation of p70S6K at Thr229 and inactivation of 4EBP1^63^. Activated p70S6K subsequently phosphorylates eIF4B to initiate protein synthesis^64^. 4E-BP1 also releases inhibition on eIF4E to enhance protein synthesis 65,66. Furthermore, mTORC1, similar to yeast TOR, phosphorylates mammalian ULK1, ATG13, and FIP200 complex^67^,^68^, thereby inhibiting ULK1 and ULK2 kinase activity by phosphorylating ULK1 at Ser758. Then, the activated ULK1 phosphorylates Beclin-1 at Ser14, which is necessary to induce autophagy^49^,^67^,^69^-^72^.

## HBV infection and mTOR

**Figure 3:**
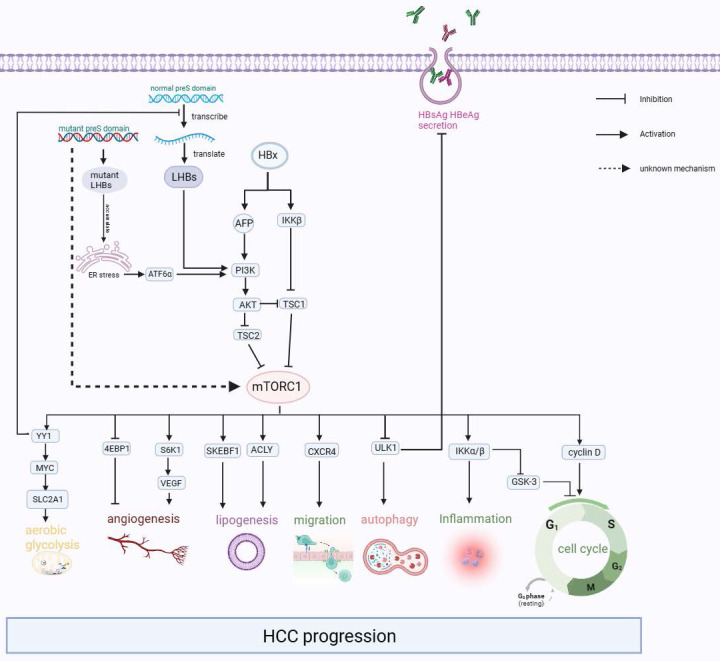
The Interaction between mTOR pathway and HCC. HBx activates the PI3K/AKT/mTOR signaling pathway by increasing the expression of AFP and activating IKKβ, which encourages malignant transformation. Besides, the accumulation of mutant L-HBsAg and excessive L-HBsAg could also activate the mTOR signaling pathway. Then the activated mTOR promotes carcinogenic-related life activities, such as aerobic glycolysis, angiogenesis, lipogenesis, migration, inflammation, and cell cycle.

After the HBV particles enter liver cells through receptor sodium taurocholate cotransporting polypeptide (NTCP) and heparan sulfate proteoglycan (HSPG)^73^,^74^, they undergo uncoating in the nucleus, and relaxed circular DNA (rcDNA) is released from the nucleocapsid into the liver nucleus. The rcDNA is converted into the template cccDNA^75^,^76^, which is transcribed and then translated to L-HBsAg, M-HBsAg, S-HBsAg, HBx proteins, HBeAg, and core proteins^77^. In some cases, these viral proteins can affect the normal physiological activities of liver cells, resulting in ER stress, activating mTOR, and ultimately leading to the progression of liver-related diseases such as cirrhosis and liver cancer (Figure 3).

### HBx protein activate mTOR

The HBV genome consists of four overlapping open reading frames (ORFs). One of these, ORFs X, encodes the small non-structural regulatory HBV X protein (HBx). HBx is composed of 154 amino acids and has a molecular mass of about 17.5 kDa^78^. The multifunctional HBx protein interacts with several host factors to affect cellular signal transduction pathways, transcriptional regulation, cell cycle progression, DNA repair, apoptosis, and genetic stability^79^.

Early research found that in vitro cell experiments conducted on Chinese Hamster Lung cells showed that, compared to the control group, cells transfected with HBx exhibited significantly higher PI3K and Akt activities^80^. However, this study did not use the liver cell line. Subsequently, experiments on human liver cancer cell lines and HBx-transgenic mice have demonstrated that overexpression of HBx increases the level of phospho-S6K1, which is downstream of mTOR^81^.

Further studies suggested that the expression of p-mTOR and its upstream p-AKT was significantly upregulated in liver-derived cells after transfection with HBx^82^-^84^. This upregulation leads to increased cell proliferation, which is also linked to inflammation and tumor angiogenesis. The occurrence of this result is based on the following clarified mechanisms: (1) HBx inhibits TSC1 and then activates mTOR through the IκB kinase (IKK) complex subunit β (IKK-β), ultimately promoting cell proliferation and new vessel formation by enhancing vascular endothelial growth factor A (VEGF-A), which promotes malignant transformation of liver cells^81^,^85^. (2) HBx prevented hepatocyte apoptosis and accelerated the cell cycle from the G1 phase to the S phase by increasing the expression of cyclinD1 through the Akt/mTOR signaling pathway^82^,^83^,^86^-^88^. (3) HBX induced Alpha-fetoprotein (AFP) expression to activate the PI3K/AKT/mTOR signaling pathway by binding PTEN with AFP, and then p-mTOR (Ser2448) enhanced HIF-1α binding to the promoters of Src, C-X-C chemokine receptor 4 (CXCR4), and Ras genes, which are oncogenes^89^. Or p-mTOR (Ser2448) promotes CXCR4 expression through binding to CXCR4 gene promoter elements directly^90^. Finally, overexpression of these oncogenes promotes invasion and metastasis in hepatocytes^91^-^95^. To summarize, it can be observed that mTOR signaling plays an important role as a molecular regulatory factor in connecting metabolic disorders and cancer in chronic HBV infection (Figure 3).

### HBsAg activates mTOR

According to the research, accumulating wild-type and mutant HBsAg can cause ER stress and turn on the Akt/mTOR signaling pathway to induce cell transformation and inflammation^96^-^99^. Particularly, the overexpression of large hepatitis B surface antigen (L-HBsAg) may participate in HBV-related hepatocarcinogenesis by activating the PI3K/Akt/mTOR pathway^100^. Besides, overexpression of small hepatitis B surface antigen (S-HBsAg) cannot change the phosphorylation levels of mTOR^101^. Additionally, numerous studies have shown that mutations in the pre-S region are associated with the formation of liver cancer through the mediating mTOR signaling pathway, which is situated within the coding region of L-HBsAg 97,102-110.

Firstly, pre-S1/2 deletion mutants (pre-S1: nt 3040-3111, pre-S2 mutant: nt 4-57) induced the enhanced expression of p-Akt and p-mTOR in HuH-7 cells^97^. The pre-S2 deletion mutant-induced mTOR activation signal cascade can not only promote lipogenesis by activating key regulators of lipid metabolism, such as sterol regulatory element binding transcription factor 1 (SREBF1) and ATP citrate lyase (ACLY), but also stimulate cell proliferation, both of which may lead to the occurrence of HCC^108^. Besides, pre-S2 deletion mutants may activate mTOR/Yin Yang 1(YY1) /myelocytomatosis oncogene (MYC) signaling to upregulate SLC2A1, which would sustain high activation rates of aerobic glycolysis and lead to tumorigenesis^109^,^110^. Interestingly, Teng et al. indicated that some pre-S1 deletions and site mutants can activate mTOR in HuH-7 cells. In turn, the up-regulated mTOR inhibited L-HBsAg synthesis at the transcriptional stage through the transcription factor YY1, which binds to the preS1 promoter (nt 2812-2816)^102^.

### mTOR affects the transcription and replication of HBV

mTOR inhibits HBV transcription and replication. Related studies have shown that constitutively active Akt1 significantly suppressed HBV RNA transcription, which in turn decreased HBV DNA replication. Given that the mTOR inhibitor rapamycin reversed this decrease in HBV gene transcription, it appears that mTOR activation was the cause^111^. Besides, treatment with mTOR inhibitors (rapamycin) on HepG2.2.15 cells increased the transcription of 3.5-kb and 2.4-kb viral RNA, the replication of HBV DNA within the cell, and the secretion of HBeAg and HBsAg 111-113. In addition, when using small interfering RNA (siRNA) specific to Akt and mTOR, similar to the use of chemical inhibitors, HBV replication and secretion of HBsAg and HBeAg were also significantly increased^112^,^113^. Another study found that these inhibitors enhance HBV replication and transcription in an HBx-dependent manner^114^. Furthermore, in HBV-infected dHepaRG cells, prolonged treatment with PI3K-AKT inhibitors also increased the extracellular HBV DNA^114^.

The specific physiological mechanism impacting the replication of HBV following the activation of the Akt/mTOR signaling pathway is based on autophagy^112^,^113^,^115^. Studies have shown that inhibiting the mTOR/ULK1 signaling pathway promotes autophagy, thereby upregulating HBV replication^113^,^116^. Conversely, knocking down the downstream effector ULK1 results in a significant decrease in HBV replication as well as HBsAg and HBeAg secretion in HepG2.2.15 cells. ULK1, as a classic autophagy mediator, is essential for the initiation of autophagosome formation^117^,^118^. and silencing ULK1 significantly reduces the frequency of autophagic puncta^113^. The formation of autophagic cells is crucial for the effective replication of HBV in various cells and animal models during HBV infection^101^,^115^,^116^,^119^-^123^. If autophagy is suppressed, there is only a slight reduction in HBV RNA levels and pgRNA packaging. However, it significantly inhibits HBV DNA replication. This indicates autophagy primarily enhances HBV replication during the process of viral DNA replication^115^. In turn, HBV can induce the formation of autophagic lysosomes in liver cells, as supported by in vitro cell experiments, animal experiments using HBV transgenic mice, and clinical pathology. It is worth noting that HBV enhances autophagy flux without increasing the degradation rate of autophagic proteins, suggesting a positive role of autophagy in HBV DNA replication^115^.

## Non-viral HCC and mTOR

The mTOR signaling pathway is frequently dysregulated in cancer and metabolic diseases^124^. Constitutive activation of the mTOR pathway may cause diet-independent HCC^125^. A comprehensive microarray study on a large number of human hepatocellular carcinoma patients shows that activation of Akt1 is one of the most consistent characteristics of HBV-induced HCC^126^. Inhibition of the PI3K/Akt/mTOR pathway can induce apoptosis and autophagy in hepatocellular carcinoma cells^127^. The previous mentioned evidence has shown that HBV infection can regulate the activity of the mTOR signaling pathway through different mechanisms, thus affecting the occurrence and development of HCC. However, some non-HBV infection factors can also lead to the activation of mTOR, which in turn leads to the occurrence of HCC (Figure 3).

Due to the liver being an organ for fat metabolism, the impact of mTOR on the development of HCC through lipid metabolism cannot be ignored in the special microenvironment of hepatocytes. The process of aliphatic acid production by hepatocytes is mediated by insulin through the PI3K/AKT/mTOR signaling pathway. Imbalance in mTOR pathway can lead to the production of lipid synthesis intermediates, which can ultimately result in steatosis and cancer^128^,^129^. The enhancement of lipid synthesis leads to the pathological accumulation of fatty acids(FA), thereby promoting inflammation and contributing to tumor progression^130^. It is found that mTORC2 can regulate the expression of certain key lipid synthesis genes at the transcriptional level^128^. However, on the contrary, another study suggested that the imbalance in the mTOR pathway is associated with cancer, rather than the development of liver steatosis^125^. In addition to lipid synthesis, mTOR can also regulate lipid lipolysis and the mobilization of lipid storage in liver cells by regulating autophagy and lysosomes^131^-^133^.

## mTOR inhibitor treatment in HCC

Due to the abnormal activation of the mTOR pathway in 40% to 50% of HCC patients^134^-^136^, there is growing interest in developing HCC treatment strategies that target mTOR. First generation mTOR inhibitors are Rapamycin (also known as Sirolimus) and its derivatives, targeting mTOR and FKBP12 to directly inhibit mTORC1. Derivatives include medications such as Temsirolimus (CCI-779), Deforolimus (AP23573), and Everolimus (RAD001)^137^. Some of them have been approved by the FDA for the treatment of advanced renal cell carcinoma or neuroendocrine tumors. For example, Sirolimus has been approved by the FDA for reducing organ rejection in patients undergoing renal transplantation in 1999^138^-^140^, and temsirolimus has been approved by the FDA for patients with advanced renal cell carcinoma (RCC) in 2007^141^. Besides, Everolimus was approved by the FDA for the treatment of adult patients with specific pulmonary and gastrointestinal neuroendocrine tumors in 2016^142^,^143^. Although the second and third generations of mTOR inhibitors have been developed, the majority of current clinical trials related to HCC still choose the first generation for research. Recent years, mTOR inhibitors have shown effectiveness in inhibiting the growth of HCC cells in both cell and animal experiments^144^-^146^. However, clinical trials conducted to test the efficacy of Rapalogs in treating HCC showed mixed results (Table 1).

For patients with advanced-stage HCC, the first-line treatment drug is the multi-kinase inhibitor Sorafenib^147^. However, when combined with Everolimus, there is no improved efficacy compared to Sorafenib alone^148^. Additionally, using single-agent Sirolimus was found to be modestly beneficial for patients. While Everolimus combined with the best supportive care did not improve survival compared to the placebo group^149^,^150^. Except for Everolimus and Sirolimus, Termosimox also fails to achieve the targeted progression free survival (PFS) endpoint of HCC^151^. However, intravenous injection of Termosimox showed a higher response in HCC compared to previously reported systemic Prescription^152^. Despite compelling preclinical evidence and scientific justification^20^,^22^,^23^,^144^,^146^,^153^,^154^, mTOR inhibitors demonstrated only modest efficacy in advanced HCC patients.

Owing to the limited effectiveness of Rapalogs as a standalone therapy for HCC, numerous clinical trials have been conducted or are currently underway to evaluate their therapeutic efficacy in combination therapies. Temsirolimus in combination with Bevacizumab increased the objective response rate (ORR) and overall survival (OS) in this population with advanced HCC^155^, which is promising but calls for more research at a more optimal dose and timing. The therapeutic efficacy of Sirolimus/ Bevacizumab doublet for advanced HCC also showed evidence of anti-vascular activity^156^. The differences in the effectiveness of mTOR inhibitors in single and combination therapies may be due to the complexity of the mTOR pathway and the heterogeneity of liver tumors.

mTOR inhibitors are not only effective in treating advanced HCC but also demonstrate impressive efficacy following liver transplantation for HCC. In the clinical trials of Sirolimus as adjuvant therapy after liver transplantation for HCC, it significantly prolonged OS and reduced the risk of death. Significantly, the benefits of Sirolimus in the initial 3-5 years of recurrence free survival (RFS) and OS are more pronounced in low-risk patients compared to high-risk groups^157^. Moreover, subgroups with AFP≥10ng/ml benefited mostly from sirolimus treatment, which improved OS, disease free survival (DFS) and HCC recurrence^158^,^159^. Therefore, focusing on the subgroups of HCC patients in clinical trials of mTOR inhibitors may be more promising.

The above clinical experiments have shown that the success of raptalogs in the treatment of HCC is not significant, and the limited therapeutic effect may be due to the following reasons: (1) mTORC1 inhibition leads to AKT activation through a negative feedback loop stemming from S6K1 or upregulating the insulin-like growth factor-1 receptor (IGF-R1)^160^,^161^. Therefore, it may actually reduce the anticancer activity of rapalogs^162^. (2) Raptalogs mainly inhibits its substrate S6K, which is related to cell proliferation. But another key substrate, 4E-BP1, has not been fully blocked^163^. (3) Patients exhibit rapamycin resistance mutations in the FRB domain of mTOR after or before treatment 164,165. Furthermore, elevated amounts of the antiapoptotic proteins Bcl-2, survivin, or Bcl-XL have resulted in rapalog resistance^166^-^169^. Therefore, genetic testing can be performed before medication to prevent rapalog resistance from delaying treatment.

In addition, mTORC1 can also be inhibited by acyclic nucleoside phosphonates (ANPs) in vitro, which are a kind of nucleoside analogue for clinical use of HBV infection. Studies have shown that ANPs reduce IL-10 production by inhibiting mTOR, and downregulation of IL-10 may promote HBV clearance in vivo by restoring the function of T cells and NK cells 170-172. Can nucleoside analogues contribute to inhibit HCC development? However, in another article, activation of mTOR was found in the liver tissue of patients taking the same type of ANP (ADV or TDF)^173^. Considering the presence of two complexes of mTOR, further research is needed on the specific effects of nucleoside analogues on mTOR and HCC progression.

## Summary and Outlook

This review has illustrated the pivotal role of mTOR in the nexus of nutrition, growth, aging, and disease, particularly in the replication of HBV particles at the level of DNA replication, RNA transcription, and antigen secretion. Simultaneously, activated mTOR, serving as an important carcinogenic pathway, also exhibits intricate interactions in HBV-related and non-viral HCC. Some studies suggest that the pathogenesis of HBV is attributed to the high-level replication of the virus, as the continuous accumulation of transcription templates will lead to the damage of infected cells. This elevated level of replication will lead to the consumption of cellular resources, such as synthetic cell membranes, lipoproteins, phospholipids, etc^182^. Additionally, mTOR is the central regulatory factor for nutrition and growth. Therefore, in the pathogenesis of HBV, mTOR is likely to have special regulation. The activation of mTOR signaling pathway during HBV-related tumorigenesis negatively regulates HBV replication and surface antigen synthesis. Therefore, the decrease of HBsAg and HBV DNA levels in serum or liver cells may not necessarily represent favorable disease improvement during the natural course of HCC, but on the contrary, it may suggest that the disease is developing toward a tumor, especially in the late stage of the disease. Considering the important role of the mTOR signaling pathway in various types of cancer^183^-^185^, currently, many rapamycin analogs, such as Everolimus and Temsirolimus, have been approved by the Food and Drug Administration^186^, Previous studies reported the importance of completely inhibiting mTORC1 effectors (RPS6 and eIF4E) in hepatocarcinogenesis^187^. In addition to treating HCC, mTOR inhibitors have also been found to be effective in reducing HCC recurrence after liver transplantation^188^. Moreover, the study of mTOR and its impact on the life cycle of HBV is valuable for the development of safe and effective therapies against viral effects. Although there have been many studies on mTOR signaling pathway in carcinogenesis, the unique characteristics of liver cells and the particularity of the HBV life cycle make the exploration of mTOR in HBV-related HCC both meaningful and worthy of further investigation.

## Funding

This work was supported by the grants from the National Natural Science Foundation of China (No. 82202612) and the Natural Science Foundation of Beijing Municipal (No.7232142).

## Figures and Tables

**Table 1:** Summary of completed clinical trials with mTOR inhibitors in HCC

	Drug	Trail Phase	Number of enrolled patients	HCC Stage	Child-Pugh Score	Study design	Countries And Regions	Result	ID
Adjuvant therapy after liver transplantation	Sirolimus vs mTOR inhibitor free	III	510	Milan criteria and extended	NA	Randomized	global multicenter	Completed^157^,^158^,^174^. Sirolimus prolonged OS and reduced the risk of death.	NCT00355862
		III	397	Exceeding the Milan criteria	NA	Non-randomized	China	Not yet completed^175^.	ChiCTR2100042869
	Sirolimus vs Tacrolimus	II	45	Exceeding the Milan criteria	NA	Randomized	Korea	Completed 159. While sirolimus prolongs OS, it does not reduce HCC recurrence.	NCT01374750
		III	220	Exceeding the Milan criteria	NA	Randomized	China	Recruiting.	NCT00554125
Adjuvant therapy after TACE	TACE +/- Everolimus	I/II	27	Intermediate stage B	A, B (<8)	Randomized	Switzerland multicenter	The study was terminated due to low enrollment.	NCT01009801
		II	65	Intermediate stage B	A, early B	Randomized	Asia, multicenter	This study was terminated due to low enrollment.	NCT01379521
Single agent for advanced HCC	Sirolimus	pilot	21	I to IV (TNM)	A/B/C	Non-randomized	Sweden	Completed^176^. A temporary disease-control rate (PR + SD) was observed.	NA
		II	25	B,C (BCLC)	A/B	Non-randomized	France	Completed 177. Sirolimus shows antitumoural efficacy.	Sirolimus
		pilot	18	B,C,D (BCLC)	A/B/C	Non-randomized	Austria	Completed 150. Sirolimus shows minimal effectiveness in patients with liver cirrhosis and advanced HCC.	NA
	Temsirolimus	II	45	advanced	A	Non-randomized	Hong Kong, China	Completed 151. The targeted PFS endpoint was not reached.	NCT00321594
		II	25	advanced	A/B	NA	America	Completed 152. Temsirolimus showed higher responses than previous report.	NCT01567930
	Everolimus	I/II	28	B,C (BCLC)	A/B	Non-randomized	America	Completed^178^. Everolimus was observed initial anticancer activity.	NA
		I/II	39	advanced	A, B(≤9)	Randomized	Taiwan, China	Completed^179^. This study recommends that future HCC studies on the dosage of everolimus should be conducted at a dose of 7.5mg/day.	NCT00390195
		III	546	advanced	A	Randomized	global multicenter	Completed 149. The OS of patients with HCC was not improved by everolimus.	NCT01035229
Adjuvant combination therapy for advanced HCC	Sorafenib and Temsirolimus	I	25	III, IV	A, B (≤7)	Non-randomized	America multicenter	Completed^180^. The maximum-tolerated dose (MTD) was sorafenib 200 mg twice daily plus temsirolimus 10mg/week.	NA
		I	25	III, IV (AJCC)	A, B (≤7)	Non-randomized	America multicenter	Not yet published.	NCT01008917
		II	29	II,III,IV(AJCC)	A, B (≤7)	Non-randomized	America multicenter	Not yet published.	NCT01687673
	Sorafenib and Everolimus	II	30	advanced	A	Randomized	America	Completed 181. The MTD was sorafenib 400 mg twice daily plus everolimus 2.5mg/day.	NA
		II	106	B,C (BCLC)	A, B (≤7)	Randomized	global multicenter	Completed^148^. Sorafenib and Everolimus combination failed to improve the efficiency compared to Sorafenib solely.	NCT01005199
	Temsirolimus + Bevacizumab	II	28	advanced	A	NA	Canada	Completed 155. The overall response rate and median OS have both improved under Temsirolimus and Bevacizumab combination therapy.	NCT01010126
	Everolimus+ Bevacizumab	II	36	B,C (BCLC)	A/B	randomized	Germany multicenter	Not yet published.	NCT00775073
	Sirolimus + Bevacizumab	II	27	advanced	A/B	Non-randomized	Singapore	Completed^156^. The recommended dose was bevacizumab 5mg/kg /14 days and rapamycin 4 mg/day.	NCT00467194
	Everolimus and Pasireotide	II	24	C (BCLC)	A(≤6)	randomized	America multicenter	Not yet published.	NCT01488487
